# Improving the Yield of Feruloyl Oligosaccharides from Rice Bran through Enzymatic Extrusion and Its Mechanism

**DOI:** 10.3390/foods12071369

**Published:** 2023-03-23

**Authors:** Fenghong Deng, Xiuting Hu, Yueru Wang, Shunjing Luo, Chengmei Liu

**Affiliations:** The State Key Laboratory of Food Science and Technology, Nanchang University, Nanchang 330047, China

**Keywords:** rice bran, feruloyl oligosaccharides, enzymatic extrusion, yield

## Abstract

Rice bran, rich in feruloyl arabinoxylan, is a good source of feruloyl oligosaccharides (FOs). To prepare FOs, bran was often hydrolyzed by amylase and protease to remove starch and protein and then hydrolyzed by xylanase, which was time-consuming and had a low yield. To solve the above problems, enzymatic extrusion was used to treat rice bran, and the effects of traditional hydrolysis, a combination of traditional extrusion and hydrolysis (extrusion-hydrolysis) and enzymatic extrusion on the yield of FOs were investigated and compared in this study. It was found that traditional extrusion and enzymatic extrusion significantly increased the yield of FOs. Particularly, the yield of FOs resulting from enzymatic extrusion was increased to 5.78%, while the yield from traditional hydrolysis was 4.23%. Microscopy analysis showed that extrusion damaged the cell wall of bran, which might increase the accessibility of xylanase to arabinoxylan and the yield of FOs. Spectroscopy analysis suggested that FOs obtained by different pretreatments had similar structures. It was obvious that enzymatic extrusion saved the time for removal of starch and protein and increased the yield of FOs. In addition, the highest yield of FOs was found at the moisture content of 30% and the screw speed of 50 rpm. This study provided an efficient method for the preparation of FOs that is suitable for industrial production.

## 1. Introduction

Cereals, the staple food of human beings, are consumed in huge amounts each year. Particularly, China produces and consumes the most rice in the world. As a result, rice bran, a by-product during rice processing, is produced in huge quantities. Currently, most rice bran is used as livestock feed, and only a small amount is made into edible bran for commercial sale, resulting in a low value. Rice bran is rich in non-starch polysaccharides, mainly hemicellulose, including arabinoxylan (AX) and β-glucan, as well as cellulose and lignin [1]. Among these non-starch polysaccharides, AX is an important dietary fiber. AX is one component of the cell wall of rice bran, accounting for 7% (*w*/*w*) of rice bran, and plays an important role in regulating gut microbiota [2]. Moreover, partial hydrolysis of AX produces feruloyl oligosaccharides (FOs), which are recognized as functional food ingredients with synergistic physiological activities of oligosaccharides and ferulic acid by the United States Food and Drug Administration (FDA) [3]. The first FO product (GRAS Notice No.343., 2010) was prepared from wheat bran and was approved by the FDA in 2010. Thus, the AX structure determines the structure of FOs to a great extent. AX is composed of a backbone of D-xylopyranosyl linked by β-(1,4) glycosidic linkages and α-L-arabinofuranosyl substituents linked by α-(1,2) and/or α-(1,3) glycosidic linkages attached to the O-2 and/or O-3 position of xylopyranosyl residues [4]. Rice bran also contains a variety of phenolic compounds, such as ferulic acid, *p*-coumaric, caffeic acid and erucic acid, among which the ferulic acid content is the highest. Ferulic acid is usually etherified or esterified to the O-5 of the arabinofuranosyl residues of AX [5]. Moreover, the radical coupling reactions between feruloyl residues result in intramolecular and intermolecular crosslinking of AX molecules and the linkage between AX molecules and lignin or protein molecules [6]. Because of these structural characteristics, AXs are mostly insoluble, which limits their application. Therefore, cereal bran is generally hydrolyzed by enzymes or mild acid to prepare FOs with good solubility. Moreover, FOs have the ability to remove free radicals, promote the proliferation of probiotics, and regulate the immune function of the human body [7,8,9].

During preparation of FOs, starch and protein should be removed first, and then bran is hydrolyzed. Traditionally, starch and protein were removed by hydrolysis using amylase and protease, respectively, which was time-consuming. On the other hand, as hemicelluloses were highly branched and bridged with cellulose or lignin molecules, these complicated structures impeded the formation of enzyme-substrate intermediates, which resulted in inefficient hydrolysis of AXs [10]. Therefore, to improve the yield of FOs, bran was often pretreated by physical methods, such as microwave, ultrasound and high-pressure steaming [11,12,13].

Enzymatic extrusion is a new extrusion technology based on traditional extrusion, in which the extruder is used as an enzyme reactor to accelerate the enzymatic reaction [14]. Enzymatic extrusion is usually carried out under the conditions of high substrate concentration and high shear force. It can effectively act on complex biopolymers with a high degree of polymerization, crystallinity and structural strength, impose a porous microstructure and expose the reactive site of the enzyme [15]. Therefore, it was speculated that the extrudate from enzymatic extrusion might be more accessible to xylanase for preparation of FOs. In addition, compared with traditional hydrolysis to remove starch and protein, enzymatic extrusion might greatly shorten the time required for these steps. At present, amylase, cellulase, and protease are used in enzymatic extrusion and mainly plays a role in liquefying starch, producing bioethanol and improving dough quality, respectively [16,17,18]. However, the application of enzymatic extrusion in the pretreatment of cereal bran to prepare FOs has not been reported yet. Therefore, the purpose of this study was to increase the yield of FOs by enzymatic extrusion and investigate the related mechanism.

## 2. Materials and Methods

### 2.1. Materials

Rice bran was obtained from Hunan Hekang Ecological Agriculture Development Co., Ltd. (Yiyang, China), milled through a 40-mesh sieve, sealed in polyethylene bags, and kept at room temperature for further analysis. Thermostable α-amylase (EC 3.2.1.1, Termamyl DS SC, from *Bacillus amyloliquefaciens*) and neutral protease (EC 3.4.21.62, Neutrase 0.8 L, from *Bacillus amyloliquefaciens*) were supplied by Novozymes (Beijing, China). Amberlite XAD-2 was obtained from Rohm and Haas (Philadelphia, PA, USA). Xylanase (EC 3.2.1.8, from *Trichoderma viride*), ferulic acid, arabinose, and xylose were purchased from Aladdin Industrial Inc. (Shanghai, China). All other chemicals and solvents used were of analytical grade or above.

### 2.2. Pre-Treatment of Rice Bran

In this work, rice bran was divided into three groups as demonstrated inFigure 1 to explore the effect of enzymatic extrusion on the yield of FOs. In the control group, rice bran was pretreated by traditional hydrolysis [13]. Specifically, 200.0 g rice bran was suspended in 2.0 L distilled water, and the pH was adjusted to 6.0 by 1.0 M HCl solution. Subsequently, thermostable α-amylase (720 U/g bran) was added, and the enzymatic reaction occurred at 95 °C for 40 min. Then, the suspension was cooled to 50 °C, and the pH was adjusted to 6.5 by 1.0 M NaOH solution. Subsequently, neutral protease (1440 U/g bran) was added, which reacted at 50 °C for 4 h and was inactivated at 100 °C for 10 min. Finally, the suspension was centrifuged at 4800 rpm for 15 min to remove the supernatant.

For the combination of traditional extrusion and hydrolysis by amylase and protease (extrusion-hydrolysis), rice bran was mixed with 40% (*w*/*w*) distilled water and then extruded. The resultant extrudate was dried at 50 °C, ground, mixed with 40% (*w*/*w*) distilled water and extruded again. The extruded rice bran was dried at 50 °C, ground, and finally treated by traditional hydrolysis as described above to remove starch and protein.

Enzymatic extrusion of rice bran was carried out as follows [14]. Rice bran was mixed with 40% (*w*/*w*) distilled water containing thermostable α-amylase (720 U/g bran) and then extruded. The resultant extrudate was dried at 50 °C, ground, mixed with 40% (*w*/*w*) distilled water containing neutral protease (1440 U/g bran), and extruded again. Finally, the extruded rice bran was dried at 50 °C and ground.

Extrusion was carried out with a laboratory scale, co-rotating intermeshing twin-screw extruder (FMHE36-24, FUMACH, Changsha, China) with a barrel diameter at 36.0 mm, an L/D ratio at 24:1 and a circular die of 10.0 mm diameter. The extruder barrel was divided into five zones: the first zone, the feeding zone, was kept at room temperature, and the temperature of the subsequent four zones was adjusted according to the requirement. The extrusion parameters used in traditional extrusion and enzymatic extrusion were the same. During the first extrusion, the temperature of zone 2 to 4 was set at 95 °C, and the temperature of zone 5 was set at 105 °C to inactivate amylase. During the second extrusion, the temperature of zone 2 to 4 was kept at 50 °C, and the temperature of zone 5 was set at 60 °C to inactivate protease. The screw speed was 50 rpm, and the rate of adding water was 0.2 kg/h.

To remove the soluble substances, the precipitates after traditional hydrolysis and extrusion-hydrolysis and extrudate from enzymatic extrusion were washed three times with hot distilled water and twice with distilled water, 78% ethanol, and 95% ethanol at room temperature, respectively. Finally, the solids were dried and ground to obtain the traditional hydrolysis-induced fiber concentrate (H-FC), extrusion-hydrolysis-induced fiber concentrate (EH-FC) and enzymatic extrusion-induced fiber concentrate (E-FC).

### 2.3. Xylanase Hydrolysis of Fiber Concentrates

To prepare FOs, xylanase hydrolysis of fiber concentrates was conducted according to a previous study [13]. The above fiber concentrates (5.0 g) were dispersed into 80.0 mL of the acetate buffer (pH 5.0, 100 mmol/L), and xylanase (2000 U/g bran) was added to the suspension and reacted at 45 °C for 4 h. After the reaction, they were centrifuged to obtain the supernatant, and the supernatant volume was adjusted to 100 mL for subsequent analysis.

### 2.4. Purification of FOs

FOs were purified using column chromatography as described previously [19]. The xylanase hydrolysates were concentrated by rotary evaporation and passed through a 0.45 μm membrane. The concentrated hydrolysates were loaded onto a 2.5 × 50 cm column packed with Amberlite XAD-2, which was previously washed with 95% ethanol and distilled water. The flow rate was 1.5 mL/min, and the hydrolysates were adsorbed and equilibrated overnight. Subsequently, the column was eluted with distilled water for 2 column volumes, 50% ethanol for 3 column volumes and 95% ethanol for 2 column volumes, respectively. The elution flow rate was 2.0 mL/min. The eluates obtained by 50% ethanol were collected, concentrated, and freeze-dried to obtain purified FOs.

### 2.5. Composition Analysis

The total starch content was determined by the total starch kit from Megazyme (K-TSTA, Megazyme, Wicklow, Ireland). Since 2.0 M KOH solution was used to dissolve starch in this method, the total starch content, including the resistant starch content, was determined. The protein content was determined by Kjeldahl.

The ferulic acid content was determined by high performance liquid chromatography (HPLC) [20]. For the free ferulic acid, the hydrolysate was directly passed through a 0.45 μm membrane for HPLC analysis (1260 Infinity, Agilent, Santa Clara, CA, USA). For the total ferulic acid, 20.0 mg fiber concentrate or 1.0 mL hydrolysate was treated with 5.0 mL of 2.0 M NaOH solution for 2 h in the darkness. The reaction was stopped by adjusting the pH to 2.0 with 6.0 M HCl solution, and ferulic acid was extracted with 10.0 mL ethyl acetate by vortex for 2 min. Subsequently, the mixture was centrifuged, and 4.0 mL of the upper layer of ethyl acetate was dried under nitrogen. Finally, the dried ferulic acid was redissolved in 4.0 mL methanol and passed through a 0.45 μm membrane for HPLC analysis. A C18 column (XBridge C18, 5 μm, 4.6 × 250 mm, Waters, Milford, MA, USA) was used. The mobile phase was isocratic elution of acetonitrile and 1.0% glacial acetic acid with a 20:80 ratio, and the flow rate was 0.8 mL/min. Each sample (15.0 μL) was injected into the column and detected at 320 nm with an UV detector. The bound ferulic acid content was the total ferulic acid content minus the free ferulic acid content. The ferulic acid yield was expressed as the following equation:

(1) $$Y(\%)=\frac{M_{1}}{M_{2}}\times \;100$$

where M_1_ was the bound ferulic acid content in FOs, and M_2_ was bound ferulic acid content in fiber concentrates.

The pentose content was determined as described previously [21]. Briefly, the fiber concentrate was dispersed in 2.0 mL of distilled water. The fiber concentrate suspension or the hydrolysate (2.0 mL) was mixed with 10.0 mL of the extraction reagent and kept at 100 °C for 25 min. The extraction reagent was composed of 2.0 g phloroglucinol, 10.0 mL absolute ethanol, 110.0 mL glacial acetic acid, 2.0 mL concentrated hydrochloric acid and 1.0 mL 17.5 g/L glucose solution. As a result, the pentosan in fiber concentrates and FOs was hydrolyzed into pentose, and pentose reacted with phloroglucinol. After the reaction, the solution was quickly cooled down, and the absorbance was measured at 552 nm and 510 nm. Pentose had strong absorption at 552 nm but very low absorbance at 510 nm, while there was almost no difference between the absorbance of hexose at 552 nm and 510 nm. Therefore, to eliminate the interference of hexose, the percentage of pentose was calculated by subtraction of the absorption value at 510 nm from that at 552 nm and comparison of the results with a xylose calibration curve. The pentose yield was expressed as the following equation:

(2) $$Y\;(\%)=\frac{M_{3}}{M_{4}}\times \;100$$

where M_3_ was the pentose content in FOs and M_4_ was the pentose content in fiber concentrates.

### 2.6. Light Microscopy Analysis

Rice bran and fiber concentrates were mixed with 1.0% toluidine blue in the darkness and then washed with distilled water to remove the excess dye. The samples were embedded in the frozen section medium (NEG-50 6502Y, Thermo, Waltham, MA, USA), and slices of 10 μm thickness were made with a cryostat (CryoStar NX50 OP, Thermo) using a steel knife. The cell wall structures of rice bran and fiber concentrates were observed by a light microscope (ECLIPSE Ni, Nikon, Tokyo, Japan) equipped with a camera (DS-Fi3, Nikon, Tokyo, Japan). A 10× objective was used.

### 2.7. Scanning Electron Microscopy (SEM) Analysis

Rice bran and fiber concentrates were mounted on circular aluminum stubs with double-sided adhesive carbon tape, followed by sputtering a thin film of gold. Subsequently, the morphology was photographed by a scanning electron microscope (Quanta-200, FEI Company, Eindhoven, The Netherlands) at 2500× magnification with an accelerating voltage of 5 kV.

### 2.8. Characterization of FOs

FOs were dissolved in 3-(N-morpholinyl) propane sulfonic acid (MOPS, 100 mM, pH 6.0) buffer, and the absorption spectra were obtained by scanning in the range from 220 to 400 nm using an UV spectrophotometer (DS-11 FX+, DeNovix, Wilmington, DE, USA).

The purified FOs (1 mg) were ground with 140 mg KBr and then pressed into a transparent tablet with 1 mm thickness. The tablet was placed onto a sample rack, and the Fourier transform infrared spectroscopy (FT-IR) spectra of FOs were collected between 4000 cm^−1^ and 400 cm^−1^ on an FT-IR spectrophotometer (Nicolet 5700, Thermo, Waltham, MA, USA) with 64 scans at a resolution of 4 cm^−1^.

### 2.9. Optimization of Enzymatic Extrusion

To further improve the yield of FOs, enzymatic extrusion was carried out at different moisture contents (30% and 40%) or different screw speeds (50, 100 and 150 rpm). When the effect of moisture content on the yield of FOs was explored, the screw speed was fixed at 50 rpm, and the moisture was set at 30% or 40%. When the effect of screw speed on the yield of FOs was explored, the moisture content was fixed at 30%, and the screw speed was set at 50, 100, or 150 rpm. The pre-extrusion treatment and other extrusion conditions were the same as those used for the preparation of E-FC as described in Section 2.2.

### 2.10. Statistical Analysis

In this work, all experiments were conducted three times, and the results were expressed as the mean with standard deviation (SD). The data were analyzed by one-way analysis of variance (ANOVA) or independent samples *t*-test using SPSS software (Version 22.0). Duncan’s test was used to analyze the difference between the mean values of each experiment, and the difference was considered statistically significant if *p* < 0.05.

## 3. Results and Discussion

### 3.1. Composition of Rice Bran and Fiber Concentrates

The chemical composition of the rice bran and fiber concentrates obtained by the three above approaches is listed in Table 1. Rice bran contained 10.30% starch, 16.19% proteins, 0.46% ferulic acid and 26.09% pentose, which was in accordance with the previous study [22]. The starch content in H-FC, EH-FC and E-FC was 0.21%, 0.54% and 0.32%, respectively, which suggested that hydrolysis by amylase effectively removed starch and significantly decreased the starch content. Particularly, H-FC and E-FC had similar starch content, although the hydrolysis time was much shorter in enzymatic extrusion (~6 min). Similarly, it was found that starch was rapidly liquefied by thermostable α-amylase during enzymatic extrusion [23]. This might have been due to mechanical forces during extrusion that reduced the thermal energy needed to deconstruct starch granules, which accelerated the degradation of starch [24]. It was reported that the thermostable resistant starch that could not be degraded by amylase was formed during extrusion cooking and subsequent cooling [25], probably leading to the highest starch content in EH-FC.

The protein content in H-FC, EH-FC and E-FC was 9.07%, 10.54% and 11.64%, respectively. Obviously, the hydrolysis efficiency of protease was much lower than that of amylase during extrusion, which might have been due to the tight binding between protein molecules and lignocellulose molecules through dimerized ferulic acid bridge in rice bran [26]. Our previous experiments also proved that increasing the amount of protease during traditional hydrolysis did not further decrease the protein content (data not shown). Moreover, the residual protein content of H-FC was the lowest, which might have been due to the much longer hydrolysis time and the higher liquid–solid ratio during traditional hydrolysis. Enzymes worked more efficiently at higher moisture levels, which made it easier for enzymes to diffuse. On the other hand, during extrusion, the sugar molecules in the bran matrix might react with the protein molecules, resulting in protein molecules aggregating and forming products that were difficult to degrade [25]. Obviously, starch hydrolysis produced a large amount of reducing sugars during enzymatic extrusion that were much easier to conjugate with proteins. Thus, E-FC had the highest protein content.

No free ferulic acid was detected in fiber concentrates by HPLC, which might have been due to washing that removed free ferulic acid from fiber concentrates. The ferulic acid content in H-FC, EH-FC and E-FC was 0.71%, 0.72% and 0.78%, respectively. That is, E-FC had the highest ferulic acid content. This result was consistent with the previous report [14], which found that reducing sugars produced by starch during extrusion protected polyphenols. In addition, Xu et al. found that the phenol compounds were protected during enzymatic extrusion [27]. Obviously, the retention of ferulic acid was beneficial for the preparation of FOs.

The monosaccharide units of AXs and FOs were xylose and arabinose, which both belonged to pentose. Thus, the pentose content was also determined. The pentose content of H-FC, EH-FC and E-FC was 52.06%, 37.06% and 39.40%, respectively. That is, H-FC had the highest pentose content. As stated above, H-FC had the lowest protein content, probably resulting in the highest proportion of pentose. Moreover, part of the insoluble dietary fiber was converted to soluble dietary fiber during extrusion and removed by subsequent washing, which might have resulted in lower pentose content in the extruded rice bran.

### 3.2. The Yields of Ferulic Acid and Pentose in FOs

**Figure 2:**
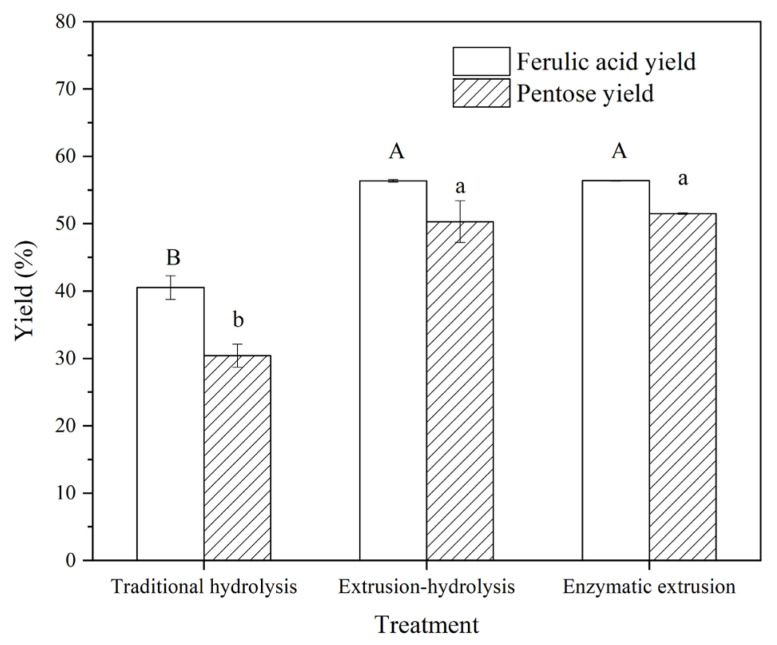
The yields of ferulic acid and pentose of FOs from traditional hydrolysis, extrusion-hydrolysis and enzymatic extrusion. The columns in figure with different letters are significantly different at *p* < 0.05, based on the Duncan’s test.

FOs were composed of ferulic acid and pentose. Thus, the yields of ferulic acid and pentose were used to characterize the yield of FOs (Figure 2). The FOs prepared from H-FC had a ferulic acid yield of 41.30% and a pentose yield of 30.40%. The ferulic acid yield of FOs from EH-FC and E-FC was increased to 56.35% and 56.38%, respectively. Moreover, the pentose yield of FOs from EH-FC and E-FC was increased to 50.29% and 51.49%, respectively. That is, E-FC and EH-FC resulted in higher yields of FOs but had no significant difference. These results indicated that compared with traditional hydrolysis, extrusion could effectively increase the yield of FOs. The previous study indicated that extrusion increased the hydrolysis efficiency of xylanase on bran, thereby increasing the solubility of AXs [28]. Moreover, the similar yields of ferulic acid and pentose of FOs from extrusion-hydrolysis and enzymatic extrusion demonstrated that the increase in the yield of FOs mainly resulted from the function of extrusion. Similarly, Dang et al. investigated effects of extrusion and xylanase-added extrusion on the yield of total soluble substances and found that the increase in total soluble substances was mainly achieved through extrusion instead of xylanase addition [22]. Thus, it was inferred that the mechanical force dominated in improving the yield of FOs during enzymatic extrusion.

In summary, H-FC had the lowest protein content and the highest pentose content, while hydrolysis of H-FC led to the lowest yield of FOs. Thus, it was inferred that the composition of bran had little effect on the yield of FOs. In general, high protein content did not always hinder xylanase from attacking AXs, and higher pentose content did not always result in a higher yield of FOs. On the other hand, FOs resulting from the above different pretreatments had a similar ratio of the pentose moiety to the ferulic acid moiety, which suggested that FOs from different pretreatments had similar molecular structures. Xylanase, an endonuclease, acted on β-1, 4 glycosidic bonds in the main chain of xylan [29]. Thus, the FOs’ structures depended mainly on the xylanase type when the same substance was used. Moreover, the structures of FOs from rice bran were similar [30,31,32]. Specifically, the backbones of FOs were composed of xylose with one or two arabinose residues substituted, and usually only one ferulic acid was attached to the arabinose residue.

### 3.3. Microstructure of Rice Bran and Fiber Concentrates

**Figure 3:**
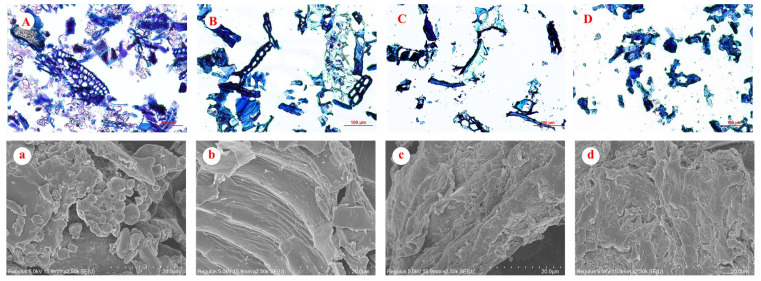
Light microscope images and SEM images of rice bran (**A**,**a**), H-FC (**B**,**b**), EH-FC (**C**,**c**) and E-FC (**D**,**d**). H-FC is traditional hydrolysis-induced fiber concentrate, EH-FC is extrusion-hydrolysis-induced fiber concentrate, and E-FC is enzymatic extrusion-induced fiber concentrate.

The cross section of the bran cell wall was observed by a light microscope (Figure 3A–D), and the surface morphology of bran was observed by SEM (Figure 3a–d). Raw rice bran had a continuous and compact cell wall structure and some unstained particles (Figure 3A), which might be starch granules. Correspondingly, there were many elliptical spherical particles on the surface (Figure 3a), which further confirmed the presence of starch granules. However, these particles were not found in three fiber concentrates, which indicated that starch was successfully removed by amylase. The H-FC still had the complete cell wall, and the cell wall was more distinct (Figure 3B). Correspondingly, the surface was smooth, tight and contiguous due to the removal of starch and protein (Figure 3b). However, the cell walls of EH-FC and E-FC were damaged, and the edges were irregular (Figure 3C,D). The surfaces became loose and crinkly (Figure 3c,d). Moreover, the cell wall in E-FC was damaged to a greater extent (Figure 3D). These results indicated that the rigid cell wall was seriously decomposed due to extrusion and a wrinkled and loose surface was formed, which might increase the accessibility of the fiber concentrate to the enzyme solution. Thus, xylanase might more easily penetrate the cell wall structure, which was conducive to improving the yield of FOs. These results suggested that traditional extrusion and enzymatic extrusion effectively improved the yield of FOs, which was mainly due to the function of the mechanical force during extrusion. Moreover, it was obvious that enzymatic extrusion saved the time used to remove starch and protein during traditional hydrolysis. Therefore, enzymatic extrusion instead of traditional extrusion was chosen for preparing FOs, and the structure of FOs from H-FC and E-FC was compared.

### 3.4. Characterization of Purified FOs from H-FC and E-FC

**Figure 4:**
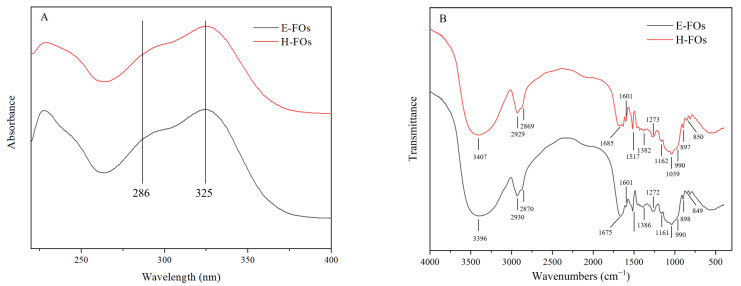
The UV (**A**) and FT-IR spectra (**B**) of H-FOs and E-FOs. H-FOs are the feruloyl oligosaccharides prepared by traditional hydrolysis and xylanase hydrolysis, and E-FOs are the feruloyl oligosaccharides prepared by enzymatic extrusion and xylanase hydrolysis.

The UV spectra of FOs prepared from H-FC and E-FC in the MOPS buffer are shown in Figure 4A. The UV spectra of H-FOs and E-FOs were similar, and the absorbance value at 325 nm was greater than that at 286 nm, which indicated that H-FOs and E-FOs had similar structures. According to a previous study [13], the absorbance of bound ferulic acid at 325 nm was greater than that at 286 nm, while the absorbance of free ferulic acid at 286 nm was greater than that at 325 nm. Thus, the above results suggested the ferulic acid existed in the bound form and the FOs were successfully prepared from H-FC and E-FC.

The FT-IR spectra of H-FOs and E-FOs were similar (Figure 4B), which further confirmed that FOs obtained from H-FC and E-FC had similar structures. As demonstrated in Table S1, the broad absorbance bands in 3600–3200 cm^−1^ in FOs were attributed to the stretching vibration of the -OH groups. The absorption bands at 2929/2930 cm^−1^ and 2869/2870 cm^−1^ in H-FOs/E-FOs resulted from the C-H antisymmetric and symmetric stretching vibration of saccharides. The absorption bands at 1382/1386 cm^−1^ in H-FOs/E-FOs were described as C-H deformation vibration. The above bands confirmed the presence of saccharides [33]. The bands at 1685/1675 cm^−1^ in H-FOs/E-FOs were attributed to the C=O stretching vibration. The absorption bands at 1162/1161 cm^−1^ and 1273/1272 cm^−1^ in H-FOs/E-FOs were ascribed to the symmetric and antisymmetric stretching vibration of C-O-C in ester group. These results indicated the presence of esterified carboxyl groups. In addition, the appearance of the bands at 1517 and 1601 cm^−1^ in FOs resulted from stretching vibration of C=C in phenyl ring [34]. Moreover, the C-O-H stretching vibration of xylopyranose led to bands at 1039/1040 cm^−1^ in H-FOs/E-FOs. The shoulder band at 990 cm^−1^ in FOs indicated the presence of the arabinose group, which was linked to xylopyranose at the O-3 position [35]. The C-H stretching vibrations of α- and β-glycosidic bonds linked to pyranose residues were confirmed by the absorption bands at 849/850 cm^−1^ and 897/898 cm^−1^ in H-FOs/E-FOs, respectively [36]. Therefore, the above characteristic bands confirmed the presence of arabinose, xylose and ferulic acid groups. That is, ferulic acid was linked to sugar by an ester bond, and FOs were successfully prepared.

The yield of purified FOs resulting from H-FC and E-FC was 4.25% and 5.79%, respectively (Table 2), which confirmed that enzymatic extrusion pretreatment significantly increased the yield of FOs. However, there was no significant difference in the ferulic acid content of H-FOs and E-FOs, which further indicated that H-FOs and E-FOs had similar structures.

### 3.5. Optimization of Enzymatic Extrusion

The above results confirmed that enzymatic extrusion improved the yield of FOs. Therefore, the enzymatic extrusion conditions were optimized. The main factors that affected properties of the extrudate included the moisture content, the extrusion temperature and the screw speed. However, the optimal temperature of α-amylase and protease was 95 °C and 50 °C, respectively. Thus, the extrusion temperature was set as their optimal temperature and not optimized. On the other hand, rice bran had a strong water absorption capacity. Thus, when the moisture content was less than 30%, rice bran fibers were likely to adhere to each other and block the screw [37]. Moreover, excessive moisture content led to lower shear force. Therefore, the moisture content was optimized in the range of 30–40%. In addition, the screw speed was optimized in the range of 50–150 rpm.

**Figure 5:**
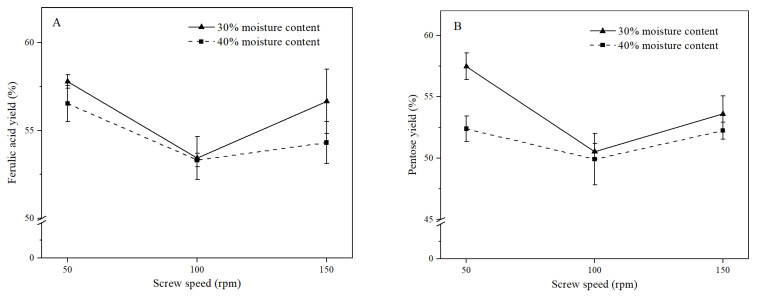
Effects of the moisture content and the screw speed on the ferulic acid yield (**A**) and pentose yield (**B**).

The moisture content had no significant effect on the ferulic acid yield when the screw speed ranged from 50 to 150 rpm. However, the pentose yield at the moisture of 30% was higher than that at 40% when the screw rate was 50 rpm (Figure 5A). This might be due to the lower moisture content that led to higher viscosity of bran and higher specific mechanical energy input from the extruder, which softened the fiber [24]. Higher mechanical energy input might be conducive to forming a loose and porous structure that facilitated penetration of the xylanase-containing solution. However, the removal of starch and proteins was more effective at the moisture content of 40% than that of 30% (Table 3), which further confirmed that the lower content of residual starch and proteins did not always result in higher efficiency of xylanase hydrolysis on rice bran.

With 30% moisture content, when the screw speed was increased from 50 to 100 rpm, the ferulic acid yield decreased from 57.80% to 53.43%, and the pentose content decreased from 57.48% to 50.53%. However, when the screw speed was further increased to 150 rpm, the yields of ferulic acid and pentose increased to 56.66% and 53.61%, respectively. A similar trend was also found in enzymatic extrusion with the 40% moisture content (Figure 5A,B). That is, the yields of ferulic acid and pentose at 100 rpm reached the lowest value, which might have been the result of a combination of residence time and mechanical force in the extruder. The lower screw speed resulted in longer residence time but lower mechanical force produced from the extruder. As the screw speed was increased, the pentose content in fiber concentrates decreased (Table 3). Similarly, Jeon et al. found that the soluble pentose content was positively correlated with the screw speed [38]. The increase in the screw speed led to higher specific mechanical energy input, which resulted in depolymerization of lignocellulose and conversion of AX chains into soluble small molecules [39]. Therefore, it was inferred that extrusion at a higher screw speed produced more soluble pentose, which was washed before xylanase hydrolysis. This might result in less pentose involved in subsequent xylanase hydrolysis and a lower yield of FOs. The lowest yield of FOs was obtained from the fiber concentrate by enzymatic extrusion at 100 rpm, which further indicated that the yield of FOs was not dependent on the pentose content in fiber concentrate but mainly due to the extent of destruction to the cell wall by extrusion. In summary, the highest yield of FOs, accompanied by the yields of ferulic acid and pentose at 57.80% and 57.48%, respectively, was obtained from enzymatic extrusion at the moisture content of 30% and screw speed of 50 rpm.

## 4. Conclusions

This work demonstrated that the yield of FOs was significantly improved by traditional extrusion and enzymatic extrusion, which was mainly ascribed to extrusion-induced damage to the cell walls. The highest yield of FOs, accompanied by the yields of ferulic acid and pentose at 57.80% and 57.48%, respectively, was obtained from enzymatic extrusion at the moisture content of 30% and screw speed of 50 rpm. Moreover, extrusion did not change the structure of FOs. Compared with the combination of traditional extrusion and hydrolysis, enzymatic extrusion reduced the time required for removal of starch and proteins by traditional hydrolysis from 270 min to 12 min. Thus, it was concluded that enzymatic extrusion was a more effective pretreatment for preparation of FOs. Recently, it was found that enzymatic conjugation with feruloylated polysaccharides can improve solubility and other properties of proteins. However, enzymatic conjugation of FOs and proteins has not been studied. It was obvious that FOs have lower molecular weight and higher solubility than feruloylated polysaccharides. Therefore, it is inferred that protein molecules may have a higher conjugation degree with FOs. In addition, the protein-oligosaccharide conjugate may have a higher protein mass ratio than the protein-polysaccharide conjugate. In summary, conjugation with FOs may be more efficient in improving the properties of proteins, which deserves to be investigated in the future.

## Figures and Tables

**Figure 1:**
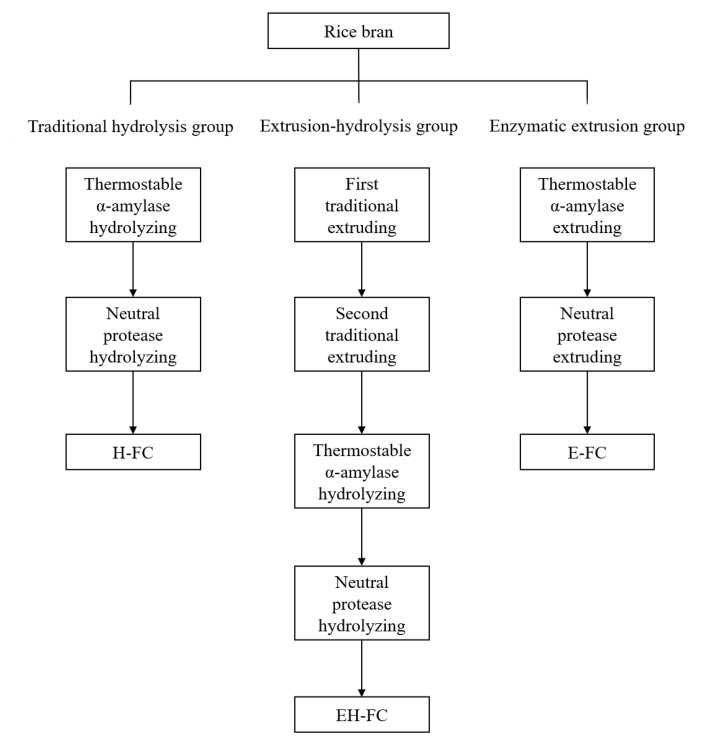
Protocol for pretreatments of rice bran to produce fiber concentrates. H-FC is traditional hydrolysis-induced fiber concentrate, EH-FC is extrusion-hydrolysis-induced fiber concentrate, and E-FC is enzymatic extrusion-induced fiber concentrate.

**Table 1:** Composition of rice bran and fiber concentrates (%, dry basis).

Samples	Starch	Protein	Ferulic Acid	Pentose
Rice bran	10.30 ± 0.14 ^a^	16.19 ± 0.21 ^a^	0.46 ± 0.02 ^c^	26.09 ± 0.64 ^d^
H-FC	0.21 ± 0.01 ^c^	9.07 ± 0.09 ^d^	0.71 ± 0.03 ^b^	52.06 ± 2.91 ^a^
EH-FC	0.54 ± 0.01 ^b^	10.54 ± 0.12 ^c^	0.72 ± 0.01 ^b^	37.06 ± 1.61 ^c^
E-FC	0.32 ± 0.03 ^c^	11.64 ± 0.18 ^b^	0.78 ± 0.02 ^a^	39.40 ± 1.97 ^b^

Each value in the table is the mean ± standard deviation of three replicates. Values in the same column with different letters are significantly different at *p* < 0.05, based on Duncan’s test. H-FC is traditional hydrolysis-induced fiber concentrate, EH-FC is extrusion-hydrolysis-induced fiber concentrate, and E-FC is enzymatic extrusion-induced fiber concentrate.

**Table 2:** The yield of purified FOs (%) and the ferulic acid content of purified FOs (%, dry basis).

Samples	The Yield of Purified FOs (%)	The Ferulic Acid Content (%)
H-FOs	4.25 ± 0.29 ^b^	7.66 ± 1.68 ^a^
E-FOs	5.79 ± 0.09 ^a^	7.54 ± 0.29 ^a^

Each value in the table is the mean ± standard deviation of three replicates. Values in the same column with different letters are significantly different at *p* < 0.05, based on independent sample *t*-test. The yield of FOs refers to the mass of purified FOs obtained from 100.0 g of fiber concentrates through xylanase hydrolysis and Amberlite XAD-2 purification. H-FOs are FOs prepared by traditional hydrolysis and xylanase hydrolysis, and E-FOs are FOs prepared by enzymatic extrusion and xylanase hydrolysis.

**Table 3:** Composition of enzymatic extruded bran fiber concentrates (%, dry basis).

Sample	Starch	Protein	Ferulic Acid	Pentose
H-FC (control)	0.21 ± 0.01 ^c^	9.07 ± 0.09 ^d^	0.71 ± 0.03 ^b^	55.43 ± 2.91 ^a^
30/50 *	0.33 ± 0.01 ^bc^	13.36 ± 0.36 ^b^	0.76 ± 0.01 ^ab^	41.38 ± 2.27 ^a^
30/100	0.39 ± 0.02 ^a^	12.69 ± 0.03 ^c^	0.72 ± 0.02 ^cd^	41.29 ± 0.66 ^a^
30/150	0.43 ± 0.01 ^a^	13.99 ± 0.02 ^a^	0.71 ± 0.03 ^d^	39.45 ± 1.77 ^ab^
40/50	0.32 ± 0.03 ^bc^	11.64 ± 0.18 ^e^	0.78 ± 0.02 ^ab^	41.42 ± 1.21 ^a^
40/100	0.31 ± 0.03 ^c^	12.31 ± 0.08 ^d^	0.80 ± 0.02 ^a^	41.17 ± 2.76 ^a^
40/150	0.35 ± 0.03 ^b^	12.89 ± 0.09 ^c^	0.75 ± 0.01 ^bc^	36.46 ± 1.66 ^b^

Each value in the table is the mean ± standard deviation of three replicates. Values in the same column with different letters are significantly different at *p* < 0.05, based on Duncan’s test. H-FC is traditional hydrolysis-induced fiber concentrate. * % moisture content (*w*/*w*)/screw speed (rpm).

## Data Availability

Data are contained within the article or Supplementary Materials.

## Supplementary Materials

The following supporting information can be downloaded at: https://www.mdpi.com/article/10.3390/foods12071369/s1, Table S1: The absorption bands of H-FOs and E-FOs and their associated functional groups on FT-IR spectra.
