# Supplementary material for: Improving the Yield of Feruloyl Oligosaccharides from Rice Bran through Enzymatic Extrusion and Its Mechanism
Source: Foods. 2023 Mar 23;12(7):1369. doi: 10.3390/foods12071369 (PMC10093099; doi:10.3390/foods12071369)
Supplement: Supplementary file 1 [file foods-12-01369-s001.zip › foods-2262012-supplementary.pdf]

Table S1. The absorption bands of H-FOs and E-FOs and their associated functional groups on FT-IR spectra.

Absorption bands of H-FOs (cm^{-1})	Absorption bands of E-FOs (cm^{-1})	Functional groups
3407	3396	Stretching vibration of the -OH groups
2869	2870	Antisymmetric stretching vibration of C-H
2929	2930	Symmetric stretching vibration of C-H
1685	1665	Stretching vibration of C=O
1601 and 1517	1601 and 1517	Stretching vibration of C=C in phenyl ring
1382	1386	Deformation vibration of C-H
1273	1272	Antisymmetric stretching vibration of C-O-C
1162	1161	Symmetric stretching vibration of C-O-C
1039	1040	Stretching vibration of C-O-H
990	990	The arabinose group
897	898	The β-(1, 4) glycosidic bond
850	849	The α-(1, 4) glycosidic bond
